# Comparison of Eosinophil Counts in Inflammatory Conditions: Multisystem Inflammatory Syndrome in Children, Kawasaki Disease, and Infectious Mononucleosis

**DOI:** 10.3390/children11020204

**Published:** 2024-02-05

**Authors:** Erdal Sarı, Özlem Erdede

**Affiliations:** Department of Pediatrics, Zeynep Kamil Maternity and Children’s Disease Training and Research Hospital, University of Health Sciences, Istanbul 34668, Turkey; ozlem.erdede@saglik.gov.tr

**Keywords:** cardiac involvement, eosinopenia, infectious mononucleosis, inflammatory disease, Kawasaki disease, MIS-C associated with COVID-19

## Abstract

This study examined the distinctions between multisystem inflammatory syndrome associated with coronavirus disease 2019, Kawasaki disease, and infectious mononucleosis. These three inflammatory disorders have commonalities according to clinical and laboratory results, particularly in relation to eosinophil levels. In this retrospective, single-center study, we documented the examination records (acute phase reactants and complete blood count) and clinical and cardiological findings of 130 patients diagnosed with multisystem inflammatory syndrome, Kawasaki disease, and infectious mononucleosis. These patients were treated and received follow-up care in our hospital from March 12, 2020, to September 13, 2022, as per the hospital records. Statistical analyses were performed using NCSS 2007, version 1 software. Eosinopenia was more prevalent in children with multisystem inflammatory syndrome than in those with Kawasaki disease, who showed normal or elevated eosinophil counts. The eosinophil counts in patients with infectious mononucleosis typically fell within the normal range. Our study found no correlation between the eosinophil counts and cardiac involvement in pediatric patients with either condition. These findings indicate a higher prevalence of eosinopenia in patients with multisystem inflammatory syndrome, irrespective of cardiac involvement, than in those with Kawasaki disease. Despite similarities in clinical findings, Kawasaki disease and multisystem inflammatory syndrome in children necessitate further studies for distinct characteristic elucidation.

## 1. Introduction

Kawasaki disease (KD) is a systemic inflammatory disease predominantly affecting children aged <5 years [1]. Moreover, it is a significant cause of acquired heart disease in these patients, primarily impacting the coronary vessels [1]. According to a prior study, its diagnosis typically relies on the presence of the following four characteristic features: oral mucosal findings, conjunctival congestion, lymphadenopathy, extremity edema, and rashes accompanying fever lasting >5 days [2]. Moreover, cases with findings that do not meet the criteria for classical KD are categorized as those of atypical KD [3]. Presently, KD management includes administering intravenous immunoglobulin (IVIG) and high-dose aspirin, supportive treatment, and addressing complications [2].

The illness known as coronavirus disease 2019 (COVID-19) is caused by the severe acute respiratory syndrome coronavirus 2 (SARS-CoV-2). Research indicates that youngsters tend to exhibit less severe symptoms when infected with this virus than adults [3,4]. In April 2020, a multisystem inflammatory syndrome in children (MIS-C) was reported in the United Kingdom (UK), Italy, and other parts of the world [5,6,7]. Several clinical features of this condition were similar to those of KD, and it was referred to as COVID-19-associated MIS-C. MIS-C is characterized by several common features, including the age group of pediatric patients, the presence of persistent fever, laboratory results indicating inflammation, clinical manifestations indicative of systemic organ dysfunction, the absence of any alternative diagnosis, and a temporal relationship with either exposure to COVID-19 or the absence of such exposure [8]. Following the initial reports from the United States of America (USA) and Europe, the World Health Organization, UK Royal College of Paediatrics and Child Health, and Center for Disease Control (CDC) have published criteria for diagnosing MIS-C [9,10,11]. Furthermore, reports pertaining to MIS-C presentations that have a similar spectrum have also been released in South Africa, India, and Pakistan [12,13,14]. Notably, a report of eight cases published in Lahore, Pakistan, showed a high incidence of coronary heart disease. However, while coronary involvement was implicated in 62.5% of the cases, cardiac dysfunction was not a prominent feature in these patients [15].

The primary objectives of MIS-C therapy are generally to decrease mortality, suppress systemic inflammation, mitigate the risk of long-term sequelae, such as coronary artery aneurysms (CAAs) or permanent cardiac dysfunction, and restore systemic organ function. To achieve these goals, a stepwise approach to immunomodulatory therapy is often recommended for patients with MIS-C. Currently, IVIG and/or corticosteroids (CSs) are the first-line agents used in the management of patients with MIS-C. Moreover, studies indicate that IVIG and CSs can be used either alone or in combination as immunomodulatory drugs to treat patients with MIS-C [16,17,18].

The commonly reported differences between MIS-C and KD include the following:

(1) While KD affects children aged <5 years, MIS-C can occur at any age (the average age is 8 years) [19].
(2) Cardiac abnormalities are prevalent in MIS-C, including decreased ejection fraction and left ventricular dysfunction [18,19,20,21,22].
(3) While 25% of untreated patients diagnosed with KD have coronary disease (CAD) [23], the incidence of CAD in patients with MIS-C is lower (around 8%) [19].
(4) Gastrointestinal and neurological symptoms are more frequent in patients with MIS-C than in those with KD [15].
(5) Patients with MIS-C exhibit lower platelet counts, lower absolute lymphocyte counts, and higher C-reactive protein (CRP) levels than those with KD [24,25].
(6) Eosinophilia is more prevalent in patients with KD than in those with MIS-C, whereas the opposite is true for eosinopenia [19,26,27,28].

Infectious mononucleosis (IMN), an acute infectious disease, predominantly affects the pediatric and adolescent population and is caused by the Epstein–Barr virus (EBV).

While fever, lymphadenopathy, and herpangina are commonly observed indicators of IMN, the clinical manifestations of this condition might exhibit variability, including symptoms such as headache, weariness, rash, jaundice, and hepatosplenomegaly [29]. Disparities exist in the populations susceptible to EBV infection, with notable variations observed between industrialized and developing nations. The prevalence of EBV infection is higher among infants and young children in underdeveloped countries, whereas the prevalence is higher among older children and teenagers in industrialized countries [30]. IMN should be included in the differential diagnosis of KD and MIS-C due to elevated inflammatory marker levels and similar presentations, including high fever [31]. Treatment of IMN and its complications is predominantly supportive [32]. However, distinguishing among these three clinical conditions can be challenging, especially in children of the same age group, as inflammatory biomarker levels are elevated in all cases. Therefore, this study aimed to assess and compare the eosinophil counts and complications in each of the three diseases. Additionally, we investigated whether the eosinophil counts could serve as a distinguishing factor among these diseases. This study addresses a notable gap in the existing literature, proposing that the eosinophil count is a contributory factor in diagnosing these conditions.

## 2. Materials and Methods

**Figure 1:**
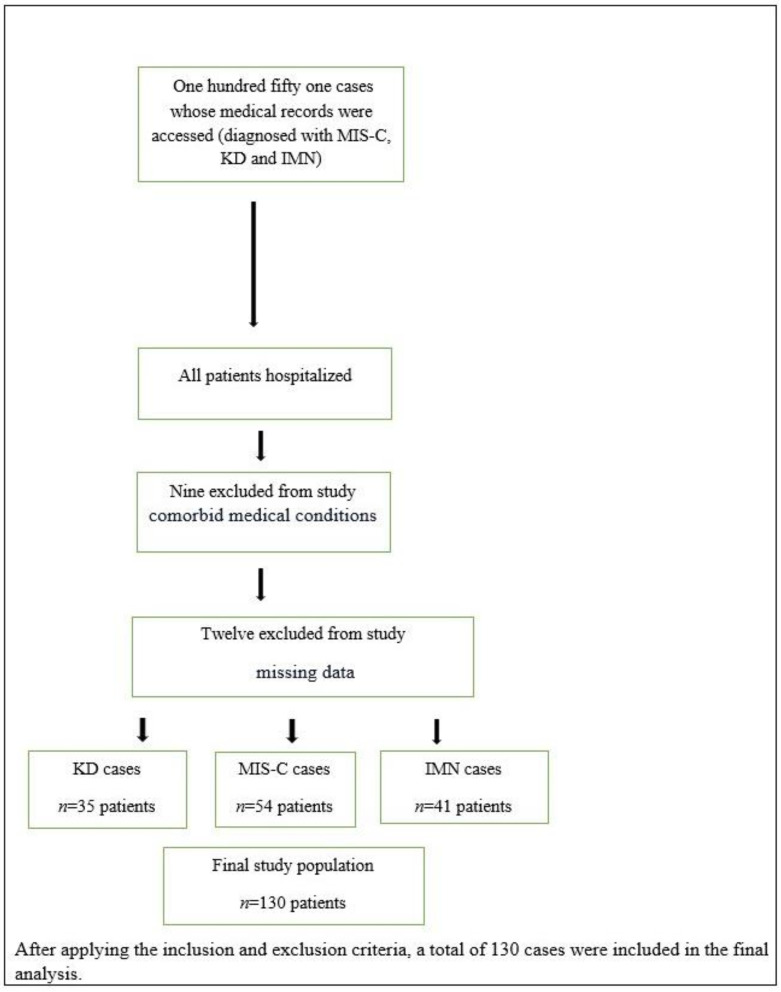
Flowchart of case identification and selection strategy. Abbreviations: IMN, infectious mononucleosis; KD, Kawasaki disease; MIS-C, multisystem inflammatory syndrome in children.

This study was conducted in a pediatric tertiary education institution located in Istanbul, Turkey, spanning from 12 March 2020 to 13 September 2022. We enrolled 130 children aged <18 years diagnosed with MIS-C, classical KD, or atypical KD and IMN. A flowchart illustrating the case identification and selection strategy is shown in Figure 1. Patient data were obtained from the hospital’s information system, including age, sex, symptoms on admission, initial examination findings, laboratory information, imaging records, pediatric infection history, and pediatric rheumatology and cardiology consultation records. 

This study adhered to the Declaration of Helsinki and was approved by the Ministry of Health of Turkey and ethics committee of our hospital (Approval number: 101; Approval date: 21 September 2022). Permission to use patient information for scientific purposes was obtained in the consent form obtained from the legal guardian (mother or father) of patients during their admission to our hospital.

### 2.1. Distinction of Diseases

Patients were categorized into classical KD, atypical KD, and MIS-C groups according to the American Heart Association [2], CDC diagnostic criteria [33], and IMN (Table 1) [34]. 

Patients who tested positive for SARS-CoV-2 antibodies (Ab) and met the diagnostic criteria in Table 1 were diagnosed with MIS-C, while those who tested negative for SARS-CoV-2 Ab and met the diagnostic criteria were considered to have KD.

We excluded patients with underlying genetic, cardiac, respiratory, or other chronic diseases; those with positive throat swab, sputum, blood, urine, and cerebrospinal fluid cultures at the time of diagnosis; and those who tested positive for viral or bacterial agents in the respiratory tract or cerebrospinal fluid. Polymerase chain reaction screening for COVID-19 was performed using reverse transcription-polymerase chain reaction of oropharyngeal and nasopharyngeal swab samples. In addition, SARS-CoV-2 Ab testing was also performed using the Elecsys^®^ anti-SARS-CoV-2 immunoassay (Roche Diagnostics, Rotkreuz, Switzerland) for the quantitative detection of Ab against the SARS-CoV-2 spike (S) protein receptor binding domain in the serum. The inclusion criteria for the IMN group are shown in Table 1. To exclude other mononucleosis-like cases, those with positive CMV immunoglobulin (Ig)M, rubella IgM, anti-human immunodeficiency virus, hepatitis B surface antigen, hepatitis A virus IgM, and toxoplasma IgM values and those with group A streptococcus detected in throat culture or rapid antigen test were excluded from this study.

The complete blood (leukocyte, granulocyte, lymphocyte, eosinophil, erythrocyte, hemoglobin, hematocrit, and platelet) count, CRP level, sedimentation rate, ferritin level, D-dimer level, and echocardiogram (at admission and during week 1) were recorded during the patients’ stay in the hospital, specifically after week 1 and month 3. Day 1 eosinophil values were intended for measurement before starting treatment with IVIG and CSs, while week 1 values were intended for assessment after treatment. The third-month values in the cases were recorded to monitor long-term findings. Eosinophil counts between 100 and 400 × 10^9^/L were considered normal, those <100 × 10^9^/L were classified as eosinopenia, and those >400 × 10^9^/L were categorized as eosinophilia. The group with cardiac parenchymal involvement consisted of patients with reduced ejection fraction and impaired left ventricular systolic performance; the group with coronary artery involvement included patients with echocardiography-detected CAD and CAA.

### 2.2. Statistical Analyses

The NCSS (Number Cruncher Statistical System) 2007 statistical software version 1 (Kaysville, UT, USA) was used for all statistical studies. In addition to descriptive statistical techniques (mean, standard deviation, median, and interquartile range) for data interpretation, the Shapiro–Wilk normality test was used to study the distribution of the variables. For intergroup comparisons of variables that did not have a normal distribution, the Kruskal–Wallis test was employed; for comparisons between subgroups and matched groups, Dunn’s multiple comparison test was utilized. To compare qualitative data, the Pearson Correlation test was utilized, along with the Mann–Whitney U test, chi-square test, and Yates-corrected chi-square test. The significance level for the results was set at *p* < 0.05.

## 3. Results

A total of 130 patients were included in this study and categorized into three groups (MIS-C, KD, and IMN). The anthropometric and laboratory data for the cases are shown in Table 2. 

Importantly, there was no substantial disparity detected in the distribution of cardiac involvement between the MIS-C and KD groups (*p* = 0.266).

Nevertheless, there was a notably greater occurrence of cardiac tissue involvement in the MIS-C group than in the KD group (*p* = 0.002). On the other hand, coronary artery involvement was more common in patients with KD (*n* = 8) than in those with MIS-C (*n* = 5), although this difference did not reach statistical significance (*p* = 0.076) (Table 3). Cardiac involvement was not examined in the IMN group.

A notable difference was observed in the mean leukocyte counts on day 1 (before treatment) across the KD, MIS-C, and EMN groups (*p* = 0.001). The average leukocyte counts on day 1 were considerably higher in the KD group than in the MIS-C and EMN groups (*p* = 0.038, *p* = 0.0001). There was no significant difference in the average leukocyte counts on day 1 between the MIS-C and EMN groups (*p* = 0.077). A notable difference was noted in the leukocyte averages over week 1 across the KD, MIS-C, and EMN groups despite the average levels falling within the normal range. This difference was significant (*p* = 0.0001). The week 1 (after treatment) leukocyte averages of the EMN group were significantly lower than those of the MIS-C and EMN groups (*p* = 0.001, *p* = 0.0001), and no significant difference was observed between the week 1 leukocyte averages of the KD and MIS-C groups (*p* = 0.450) (Table 4).

In the MIS-C group, no significant difference was observed in coronary artery involvement, cardiac parenchymal involvement, and eosinophil count (on day 1, day 7, and month 3, respectively). Similarly, no significant difference was observed in coronary artery involvement and eosinophil count (on day 1, day 7, and month 3, respectively) in the KD group (Table 5).

The eosinopenia rate at admission (87.04%) was significantly higher in the MIS-C group than in the KD and IMN groups (*p* = 0.0001). However, no significant difference was observed between the eosinophil counts of the three groups after 3 months (*p* = 0.268). It is important to notice that the MIS-C group exhibited a substantial rise in the eosinophilia rate after 3 months (46.30%) (*p* = 0.0001), whereas the KD group showed a tendency for the eosinophil counts to revert to normal compared with the initial values (*p* = 0.018). More precisely, the KD group observed a propensity for the eosinophil ratio to return to normal values by month 3 (68.57%), compared with the value documented at admission (31.43%) (Table 6).

No correlation was observed between the eosinophil counts and inflammatory marker values (CRP level, fibrinogen level, and ESR) in the entire study group, MIS-C group, KD group, and IMN group (*p* > 0.05) (Table 7).

IVIG treatment (2 g/kg iv infusion within 12 h) was applied to all MIS-C and KD cases. Additionally, steroid treatment (methylprednisolone was started parenterally at a dose of 2 mg/kg/day and then continued enterally, decreasing according to the clinical condition of the patient and ending in 3–4 weeks) was administered to 16 cases with cardiac parenchymal involvement. While the median hospital stay of patients receiving IVIG and steroid treatment was 10 days, the median hospital stay of those given only IVIG treatment was 7 days. All of our patients were discharged with full recovery. The records indicated that 3 months later, the cardiological examination findings for MIS-C and KD cases were reported as normal. In the month 3 cardiological evaluation, within the KD group, mitral insufficiency was observed in two cases with coronary artery involvement. Additionally, patent foramen ovale and right branch block (an electrocardiography finding) were identified in one case, and mitral insufficiency was detected in another case without coronary involvement. In the MIS-C group, mitral insufficiency was detected in two cases with cardiac parenchymal involvement. There were no cases of coronary findings or cardiac parenchymal involvement.

## 4. Discussion

In this study, the eosinophil counts in patients with MIS-C were significantly lower than those in patients with KD. However, no relationship was identified between the eosinophil counts and coronary artery involvement in patients with KD or between the eosinophil counts and cardiac parenchymal involvement in patients with MIS-C. Furthermore, no substantial difference in the eosinophil ratio was observed after 3 months in either disease. The eosinophil counts were elevated in 11 (31.43%) of 35 patients on day 1 and in 14 (40.00%) patients on week 1 in the KD group, decreasing to eight (22.86%) after 3 months. 

Eosinopenia was observed in 47 (87.04%) cases in the MIS-C group before IVIG treatment, decreasing to 35 (64.8%) cases after 1 week and appearing in two (3.7%) cases after 3 months. In contrast, eosinophilia was initially observed in two (3.7%) cases, increasing to 25 (46.30%) cases after 3 months. Of the 41 patients with IMN, 21 (51.22%) had normal eosinophil counts on day 1, while 33 (80.49%) reached normal eosinophil in month 3.

Generally, eosinopenia is associated with severe infections and a poor prognosis [36]. Accordingly, Gil et al. demonstrated that an inflammatory syndrome associated with eosinophil counts of <40/mm^3^ is strongly correlated with bacterial infectious diseases [37]. Similarly, several studies during the COVID-19 pandemic have found an association between eosinopenia and the severity of SARS-CoV-2 infection. For instance, Tanni et al. found that at presentation, 60% of the cases in the COVID-19 group had an eosinophil rate of 0%. Additionally, 14 of these patients exhibited zero eosinophils after 2 days, and the total number of patients in the COVID-19 group with zero eosinophils at presentation or within 2 days was 44 (88%). Meanwhile, eosinopenia was detected in 18 (86%) of the 21 patients who died in the COVID-19 group and in 13 (50%) of the 26 surviving patients [38]. However, the pathophysiology of eosinopenia in COVID-19 remains unclear. Suggested causes include redistribution of circulating eosinophils, inhibition of eosinophil production, hindered eosinophil release from the bone marrow, eosinophil apoptosis due to interferons released during acute infection, and/or chemotactic effects of increased cytokine levels [39,40].

In the literature, eosinophilia appears more prevalently in the complete blood counts of patients with KD, while eosinopenia is more frequently observed in patients with MIS-C [19,26,27,28]. Numerous studies examining eosinophils’ role in KD suggest that elevated eosinophil counts may induce allergic diseases, thus contributing to CAD. Kuo et al. reported in their study that the eosinophil count is an important factor in distinguishing KD from other febrile diseases [41]. In a study reported in 2023, eosinophils have been found to possess a relative predictive value for coronary artery dilation. It has been claimed that an increase in the eosinophil count serves as an important prognostic factor in KD [42]. Additionally, another study proposed a nomogram incorporating the eosinophil count to distinguish KD from other febrile conditions [43]. In a study by Chen et al., patients with KD exhibiting eosinophil counts above the age-appropriate mean were identified as having a higher risk of developing CAD. Conversely, those with lower eosinophil counts showed a reduced risk of CAL development. Their research specifically focused on allergic diseases associated with eosinophilia in patients with KD [26]. Terai et al. conducted a comparative analysis of peripheral blood eosinophil cell counts between 95 patients with KD and 95 age-matched febrile controls. Their findings indicated significantly higher eosinophil cell counts in patients with KD than in those of the febrile-control group (361 ± 441 vs. 65 ± 133 × 10^9^/L; *p* < 0.0001). Before treatment, eosinophilia (>350 cells/μL) was observed in 36% of patients with KD, which is markedly higher than the 4% observed in the febrile-control group (*p* < 0.0001). Additionally, eosinophilia developed within the first 2 weeks of illness in 66 (69%) patients with KD. In line with our findings, it was noted that all patients with KD who developed coronary aneurysms had eosinophilia, a sometimes evident condition [44]. In the study by Öner et al. involving 25 patients, the peripheral blood eosinophil count rate in patients with KD was significantly higher than that in the controls [45]. Moreover, Liu et al. reported that among 562 patients with KD, the eosinophil percentage (in comparison with a high leukocyte count, an elevated CRP level, a low albumin level, and an increased alanine aminotransferase level) demonstrated the highest predictive value for KD [46]. Similarly, Kuo et al. found that the eosinophil counts were elevated in the acute phase of KD, both before and after IVIG treatment. Furthermore, they observed high post-IVIG treatment eosinophil counts in patients responding well to IVIG, with a significant rise in the values during the acute phase, normalizing 3 weeks after IVIG treatment (*p* < 0.001) [47]. Notably, a report of eight cases from Lahore, Pakistan, revealed a high incidence of coronary heart disease. However, despite coronary involvement being implicated in 62.5% of cases, cardiac dysfunction was not a prominent feature in these patients [15]. A previous study associated eosinopenia and thrombocytopenia with myocardial dysfunction in patients with MIS-C. Sahoo et al. identified a strong correlation between thrombocytopenia and eosinopenia in patients with MIS-C, suggesting that clinical/laboratory parameters, such as low platelet and absolute eosinophil counts, in patients with MIS-C are indicative of mortality risk and can inform diagnostic parameters, disease severity, prognosis, treatment, and care level [28]. Cogan et al. detected CAD in a 19.9-year-old adult patient with KD; however, the eosinophil count was normal before IVIG treatment, and eosinophilia was observed 2 days after IVIG treatment [48].

In the existing literature, there was a limited amount of data on the eosinophil counts in patients with EBV-related IMN. Eosinophilia, however, was observed in both pediatric and adult cases where EBV infection was implicated [49,50]. In our study, we found no correlation between the eosinophil count and severity of inflammation in the IMN cases examined. Specifically, the eosinophil counts did not correlate with the severity of inflammation in MIS-C, KD, or IMN, and there was no significant difference between the eosinophil counts and other inflammation parameters.

This study has some limitations. First, it was a retrospective study with a limited number of patients. Second, the study cohort did not consist of cases as severe as those reported in the literature; thus, our findings cannot be generalized to all groups of patients. Further studies comprising a higher number of cases are needed to improve the generalizability of the study findings. Other limitations include the lack of distinction based on the variant periods of the COVID-19 pandemic and the absence of analysis of cases by ethnicity and demographic data. Other limitations include the lack of a control group that includes the eosinophil reference value of healthy children in our country and the lack of parasitic examinations of the cases.

## 5. Conclusions

In this study, we observed that patients with MIS-C had lower eosinophil counts, a finding more significant than other acute phase markers in distinguishing between KD and MIS-C. Moreover, our results showed that the eosinophil count was not associated with cardiac involvement. Therefore, it is unlikely that KD causes allergic manifestations related to the eosinophil count. Notably, the eosinophil count, initially low in patients with MIS-C, normalized after 3 months. We suggest that the eosinophil counts, along with other biomarker values, may be a supporting factor in the diagnosis and differentiation of MIS-C and KD. We recommend that first-line tests be performed routinely at certain times, according to the clinician’s decision, on hospitalized children.

## Figures and Tables

**Table 1:** Diagnostic criteria for classical and atypical KD, MIS-C, and IMN.

Classical KD	Patients with persistent fever for ≥5 days (or until the day of intravenous immunoglobulin administration, if administered before day 5 of fever) and exhibiting at least four of the following five clinical signs: Changes in peripheral extremities; Rash presentation; Cervical lymphadenopathy of at least 1.5 cm diameter; Changes in the oral mucosa; Bilateral conjunctival injection.
Atypical KD	Patients whose disease did not meet the abovementioned KD criteria but who had a fever and coronary artery abnormalities. The diagnostic criteria were as follows: fever persisting for ≥5 days and meeting two to three diagnostic criteria or infants presenting with idiopathic fever for ≥7 days. When the CRP level was <3 mg/dL, the ESR was <40 mm/h, and fever persisted, serial clinical and laboratory assessments were conducted. However, when peeling began, echocardiography was performed. When the CRP level was ≥3 mg/dL and the ESR was ≥40 mm/h, coupled with a positive echocardiography finding, or the presence of three or more of the following laboratory findings: (1) Anemia; (2) Albumin level of ≤3 g/dL; (3) Fever with an increased alanine aminotransferase level; (4) Platelet count of 450,000/mm^3^ after day 7 of idiopathic onset; (5) White blood cell count of ≥15,000 mm^3^; (6) If the urine white blood cell count was >10/hpf, therapy was recommended.
COVID-19-associated MIS-C	Individuals aged <21 years presenting with fever; laboratory evidence of inflammation; clinical evidence of severe illness requiring hospitalization; multisystem (≥2) organ involvement (cardiac, renal, respiratory, hematologic, gastrointestinal, dermatologic, or gastrointestinal); No plausible alternative diagnoses; and People aged <21 years who have a fever, laboratory evidence of inflammation, clinical evidence of a serious illness requiring hospitalization, multisystem (≥2) organ involvement (cardiac, renal, respiratory, hematologic, gastrointestinal, dermatologic, or gastrointestinal); who have no logical alternative diagnoses and who are positive for current or recent SARS-CoV-2 infection by reverse transcription-polymerase chain reaction, serology, or antigen test; or who were exposed to a patient with suspected or confirmed COVID-19 within 4 weeks before the onset of symptoms. Similarly, a fever of at least 38.0 °C for a full day or a report of a subjective fever lasting a full day that includes one or more of the following: elevated levels of CRP, ESR, fibrinogen, procalcitonin, D-dimer, ferritin, lactic acid dehydrogenase, or interleukin 6; low levels of albumin; and elevated counts of neutrophils, lymphocytes, and neutrophil-associated proteins. If they fit the MIS-C case definition, certain individuals who either fully or partially matched the KD criteria were reported. When evidence of SARS-CoV-2 infection was found in a juvenile mortality case, MIS-C was considered.
Infectious mononucleosis	Meeting any three of the clinical findings and any of the laboratory indicators: Fever; Angina; Cervical lymph node enlargement; Liver enlargement; Spleen enlargement; Eyelid edema. In addition, laboratory evidence of any primary EBV infection, including the following: (1) positive results for anti-EBV-capsid antigen-IgM and anti-EBV-capsid antigen-IgG antibodies and negative results for anti-EBV-nuclear antigen-IgG antibody; (2) positive results for single anti-EBV-capsid antigen-IgG antibody and low-affinity EBV-capsid antigen-IgG antibody; (3) more than four-fold increase in the dual serum anti-EBV-VCA-IgG antibody titer; (4) peripheral blood atypical lymphocyte ratio of ≥0.10 and/or lymphocytosis of ≥5.0 × 10^9^/L.

Abbreviations: CRP, C-reactive protein; EBV, Epstein–Barr virus; ESR, erythrocyte sedimentation rate; IgG, immunoglobulin G; IgM, immunoglobulin M; KD, Kawasaki disease; MIS-C, multisystem inflammatory syndrome in children; SARS-CoV-2, severe acute respiratory syndrome coronavirus 2; VCA, viral capsid antigen.

**Table 2:** Demographic and laboratory findings of the cases.

		KD Group (*n* = 35)		MIS-C Group (*n* = 54)		IMN Group (*n* = 41)		*p* Value
Age (year)	Mean ± SD	2.59 ± 2.16		6.01 ± 3.86		5.78 ± 3.65		**0.0001** ‡
Sex	Male	41	52.56%	25	51.02%	16	55.17%	0.087 +
	Female	37	47.44%	24	48.98%	13	44.83%	
Leukocyte count (10^3^/mm^3^)	Mean ± SD	15.19 ± 7.08		12.21 ± 6.86		9.59 ± 3.65		**0.001** ‡
Lymphocyte count (10^3^/mm^3^)	Mean ± SD	4.47 ± 2.75		2.4 ± 1.6		4.98 ± 2.66		**0.0001** ‡
Neutrophil count (10^3^/mm^3^)	Mean ± SD	9.34 ± 5.91		8.34 ± 5.54		3.82 ± 2.88		**0.0001** ‡
Eosinophil count (10^3^/mm^3^)	Mean ± SD	347.31 ± 393.48		58.33 ± 130.09		164.15 ± 183.08		**0.0001**
Thrombocyte count (10^3^/mm^3^)	Mean ± SD	379.71 ± 190.24		270.17 ± 133.25		277.68 ± 97.29		**0.009** ‡
CRP level (mg/L)	Mean ± SD	102.97 ± 77.1		137.27 ± 71.23		46.52 ± 44.45		**0.0001** ‡
Ferritin level (ng/mL)	Mean ± SD	319.71 ± 304.01		338.05 ± 467.68		101.33 ± 142.79		**0.004** ‡
D-dimer level (mg/dL)	Median (IQR)	1.98 (0.62–3.65)		1.62 (0.9–−3.71)		-		0.688 †
Fibrinogen level (mg/dL)	Mean ± SD	492.17 ± 129.48		560.89 ± 171.03		461.75 ± 129.6		0.097 ‡
ESR (mm/h)	Mean ± SD	65.17 ± 33.84		70.03 ± 36.24		40.98 ± 32.27		**0.001** ‡

‡ Kruskal–Wallis test. † Mann–Whitney U test + Chi-squared test. Abbreviations: CRP, C-reactive protein; ESR, erythrocyte sedimentation rate; IMN, infectious mononucleosis; IQR; interquartile range; KD, Kawasaki disease; MIS-C, multisystem inflammatory syndrome in children; SD, standard deviation.

**Table 3:** Cardiac complication rates in the MIS-C and KD groups.

		MIS-C Group		KD Group		*p* Value
Cardiac involvement (*n*, %)	Absent	34	62.96%	26	74.29%	0.266 ^a^
	Present	20	37.04%	9	25.71%	
Cardiac parenchymal involvement (*n*, %)	Absent	38	70.37%	34	97.14%	**0.002** ^a^
	Present	16	29.63%	1	2.86%	
Coronary artery involvement (*n*, %)	Absent	49	90.74%	27	77.14%	0.076 ^a^
	Present	5	9.26%	8	22.86%	

Abbreviations: MIS-C, multisystem inflammatory syndrome in children; KD, Kawasaki disease. ^a^ Chi-squared test.

**Table 4:** Leukocyte eosinophil counts in patients with MIS-C, KD, and IMN.

			KD Group	MIS-C Group	IMN Group	*p* Value ‡
Day 1 (before treatment)	Leukocyte count (10^3^/mm^3^)	Mean ± SD	15.19 ± 7.08	12.21 ± 6.86	9.59 ± 3.65	**0.001**
		Median (IQR)	14.7 (10.4–18.9)	10.99 (7.26–16.59)	7.99 (7.08–12.44)	
	Eosinophil count	Mean ± SD	347.31 ± 393.48	58.33 ± 130.09	164.15 ± 183.08	**0.0001**
	(10^3^/mm^3^)	Median (IQR)	220 (50–540)	20 (0–32.5)	100 (40–220)	
Day 7 (after treatment)	Leukocyte count	Mean ± SD	11.77 ± 3.75	13.34 ± 6.19	8.86 ± 2.59	**0.0001**
	(10^3^/mm^3^)	Median (IQR)	12.38 (8.81–13.94)	12.51 (7.97–16.87)	8.38 (7.23–10.83)	
	Eosinophil count	Mean ± SD	326 ± 310.33	127.04 ± 183.9	196.59 ± 172.52	**0.004**
	(10^3^/mm^3^)	Median (IQR)	270 (50–480)	70 (10–155)	150 (75–275)	
Month 3	Leukocyte count (10^3^/mm^3^)	Mean ± SD	10.56 ± 3.05	10.39 ± 3.41	9.53 ± 2.91	**0.304**
		Median (IQR)	9.9 (8.36–12.46)	10.16 (7.92–12.2)	8.6 (7.16–12.07)	
	Eosinophil count	Mean ± SD	293.43 ± 232.11	373.33 ± 245.31	210.73 ± 173.69	**0.268**
	(10^3^/mm^3^)	Median (IQR)	220 (130–380)	305 (147.5–592.5)	160 (95–280)	

‡ Kruskal–Wallis test. Abbreviations: IMN, infectious mononucleosis; KD, Kawasaki disease; MIS-C, multisystem inflammatory syndrome in children.

**Table 5:** Eosinophil count and cardiac complications.

	Number of Cases (*n*)		Day 1 Eosinophil Count	Day 7 Eosinophil Count	Month 3 Eosinophil Count
MIS-C group	Cardiac involvement (−) 34	Median (IQR)	20 (10-40)	70 (17.5–252.5)	350 (147.5–592.5)
	Cardiac involvement (+) 20	Median (IQR)	10 (0–20)	35 (10–80)	280 (145–587.5)
	*p* †		0.276	0.185	0.802
	Cardiac parenchymal involvement (−) 38	Median (IQR)	20 (0–40)	70 (20–212.5)	350 (157.5–600)
	Cardiac parenchymal involvement (+) 16	Median (IQR)	15 (0–20)	15 (10–77.5)	265 (132.5–532.5)
	*p* †		0.498	0.09	0.489
	Coronary artery involvement (−) 49	Median (IQR)	20 (5–35)	70 (10–185)	310 (140–585)
	Coronary artery involvement (+) 5	Median (IQR)	0 (0–140)	40 (10–95)	260 (220–695)
	*p* †		0.2	0.462	0.474
KD group	Cardiac involvement (−) 29	Median (IQR)	215 (65–532.5)	180 (45–435)	170 (145–587.5)
	Cardiac involvement (+) 9	Median (IQR)	220 (35–870)	370 (200–565)	270 (130–525)
	*p* †		0.925	0.213	0.509
	Cardiac parenchymal involvement (−) 34	Median (IQR)	2180 (47.5–532.5)	310 (65–487.5)	195 (12.5–380)
	Cardiac parenchymal involvement (+) 1	Median (IQR)	960	50	910
	*p*		-	-	-
	Coronary artery involvement (−) 27	Median (IQR)	290 (70–540)	210 (50–420)	170 (110–380)
	Coronary artery involvement (+) 8	Median (IQR)	130 (15–660)	390 (110–592.5)	260 (130–452.5)
	*p* †		0.387	0.307	0.798

Abbreviations: KD, Kawasaki disease; MIS-C, multisystem inflammatory syndrome in children; † Chi-squared test.

**Table 6:** Eosinophil ratios in patients with MIS-C and KD (comparison with published reports) [35].

Eosinophil Rate		KD Group (*n* = 35)	MIS-C Group (*n* = 54)	IMN Group (*n* = 41)	*p* Value
Day 1	Low	13 (37.4%)	47 (87.04%)	15 (36.59%)	**0.0001**
	Normal	11 (31.43%)	5 (9.26%)	21 (51.22%)	
	High	11 (31.43%)	2 (3.70%)	5 (12.20%)	
Day 7	Low	10 (28.57%)	35 (64.81%)	10 (24.39%)	**0.0001**
	Normal	11 (31.43%)	13 (24.07%)	25 (60.98%)	
	High	14 (40.00%)	6 (11.11%)	6 (14.63%)	
Month 3	Low	3 (8.57%)	2 (3.70%)	4 (9.76%)	**0.003**
	Normal	24 (68.57%)	27 (50.00%)	33 (80.49%)	
	High	8 (22.86%)	25 (46.30%)	4 (9.76%)	

Eosinophil normal range (10^9^); 1: minimum, 0.1–0.4 + Chi-squared test. Abbreviations: KD, Kawasaki disease; IMN, infectious mononucleosis; MIS-C, multisystem inflammatory syndrome in children.

**Table 7:** Correlation between eosinophil counts and other inflammatory marker values.

		Eosinophil Count			
		Day 1		Week 1	
		r *	*p* Value	r *	*p* Value
KD	CRP	0.094	0.591	−0.004	0.982
	ESR	0.321	0.089	−0.056	0.774
	Fibrinogen	0.218	0.317	−0.121	0.582
MIS-C	CRP	−0.152	0.271	−0.251	0.067
	ESR	−0.134	0.443	−0.103	0.557
	Fibrinogen	0.058	0.7	−0.045	0.764
IMN	CRP	0.195	0.223	0.101	0.531
	ESR	0.183	0.251	0.151	0.347
	Fibrinogen	0.383	0.349	0.438	0.278
Total	CRP	−0.041	0.642	−0.119	0.178
	ESR	0.14	0.153	−0.012	0.9
	Fibrinogen	0.032	0.781	−0.083	0.472

* Pearson correlation test; Abbreviations: CRP, C-reactive protein; ESR, erythrocyte sedimentation rate; KD, Kawasaki disease; IMN, infectious mononucleosis; MIS-C, multisystem inflammatory syndrome in children.

## Data Availability

The data presented in this study are available on request from the corresponding author. The data are not publicly available due to law on protection of personal data.
